# Association between blood cobalt ion concentrations and anemia and cardiovascular diseases: novel evidence of toxicity resulting from metal implants

**DOI:** 10.3389/fnut.2025.1614771

**Published:** 2025-09-03

**Authors:** Shenghao Xu, Bo Chen, Hao Wang, Xiongfeng Tang, Jianlin Xiao, Yanguo Qin

**Affiliations:** ^1^Department of Orthopedics, The Second Hospital of Jilin University, Changchun, Jilin, China; ^2^Joint International Research Laboratory of Ageing Active Strategy and Bionic Health in Northeast Asia of Ministry of Education, Jilin University, Changchun, Jilin, China; ^3^Department of Orthopedics, China-Japan Union Hospital of Jilin University, Changchun, Jilin, China

**Keywords:** metal implants, blood cobalt ion concentrations, anemia, cardiovascular disease, epidemiological evidence

## Abstract

**Background:**

Cobalt ions released from metal objects pose potential systemic toxicity risks, yet comprehensive epidemiological evidence linking blood cobalt ion concentrations with anemia and cardiovascular disease (CVD) remains limited. This study aims to investigate these associations and explore exposure thresholds.

**Methods:**

We utilized data from the National Health and Nutrition Examination Survey from 2015 to 2018. Outcomes included anemia, angina pectoris, arrhythmia, heart attack, heart failure, myocardial infarction, and stroke. We first used multivariate linear regression to demonstrate that metal implants are associated with elevated blood cobalt ion concentrations. Afterward, multivariable logistic regression, restricted cubic splines, and threshold effect analyses were applied to evaluate dose–response relationships between cobalt ion concentrations and disease.

**Results:**

Participants with metal implants exhibited 0.42 nmol/L higher blood cobalt concentrations than those without (95% confidence interval [CI]: 0.33–0.51). Each 1 nmol/L increase in cobalt was associated with a 36% higher anemia risk (adjusted odds ratio [OR]: 1.36, 95% CI: 1.31–1.41). Non-linear relationships were observed for CVD (inflection point: 3.94 nmol/L), with cobalt ion concentrations below this threshold showing stronger associations (OR: 1.27, 95% CI: 1.12–1.45). Cobalt exposure increased the risks of angina pectoris, arrhythmia, heart attack, heart failure, and stroke (all *p* < 0.05), but not myocardial infarction. Stratified analyses revealed heightened susceptibility in males.

**Conclusion:**

Metal object-derived cobalt exposure demonstrates significant dose-dependent associations with anemia and multiple CVD subtypes. These findings underscore the systemic toxicity of cobalt ions and advocate for enhanced clinical surveillance of blood cobalt levels in patients with metal implants.

## Introduction

Metal alloys, as essential biomaterials, play a crucial role in replacing and supporting aging hard tissues, significantly improving the quality of life for elderly individuals (1, 2). Implants such as artificial joints and fixation plates are widely used in the treatment of arthritis, fractures, and bone tumors, with well-established clinical utility (3–6). Among them, cobalt–chromium–molybdenum (Co–Cr–Mo) alloys have emerged as the predominant material for artificial joint prostheses due to their excellent wear resistance and biocompatibility, demonstrating substantial potential in the field of biomaterials. However, with over two million metal prosthesis implantation procedures performed globally each year, the issue of endogenous cobalt ion exposure resulting from prosthetic wear has garnered increasing attention. Studies have shown that Co–Cr alloy prostheses release cobalt ions during use, leading to elevated cobalt ion concentrations (7–14). A study involving 100 patients with metal prostheses demonstrated a significant increase in cobalt ion concentrations following metal-on-metal (MoM) total hip arthroplasty (THA) (7). Similarly, two systematic reviews reported that cobalt ion concentrations in MoM prosthesis recipients generally exceeded the normal range, with values ranging from 11.88 to 57.70 nmol/L (9, 11).

Cobalt plays both physiological and potentially toxic roles within the human body. As a central component of vitamin B12, cobalt is essential for erythropoiesis and energy metabolism (15). However, cobalt ion concentrations can induce toxicity through multiple mechanisms. Cobalt ion concentrations released from prosthetic wear enter systemic circulation and accumulate, with the hematologic system being one of the first to be affected. Studies have demonstrated that high-level cobalt exposure can lead to anemia (16). Within the hematologic system, cobalt ion concentrations contribute to anemia through two primary pathways: it inhibits hepcidin, thereby disrupting iron metabolism (17, 18), and promotes oxidative stress, leading to increased erythrocyte destruction (19, 20). Anemia is an important global public health concern, with recent data indicating an overall prevalence of 9.3% among individuals aged ≥ 2 years in the United States, reaching 13.0% in females and 5.5% in males (21). While traditional anemia research has primarily focused on nutritional deficiencies, chronic diseases, and genetic disorders, increasing attention is being directed toward the impact of environmental heavy metal exposure, particularly cobalt ion.

Cardiovascular disease (CVD) remains the leading cause of mortality worldwide, imposing a substantial social and economic burden (22). Recent data indicate that the prevalence of CVD in the United States is 11.3%, with projections suggesting an increase to 15.0% by 2050 (23). While traditional risk factors such as hypertension and dyslipidemia account for approximately 60% of the disease burden, environmental pollutant exposure has emerged as a novel risk factor warranting greater attention. Historical events, such as the 1960s “cobalt beer cardiomyopathy” epidemic (24), occupational exposure among workers in cobalt-related industries (25), and elevated cobalt ion concentrations in patients undergoing THA (26), have all highlighted the potential cardiovascular risks associated with cobalt ion exposure. A recent study by Martinez-Morata et al. (27) further supports this association, demonstrating that for every one-unit logarithmic increase in urinary cobalt ion concentration, the risk of CVD increased by 24% (HR: 1.24, 95% CI: 1.03–1.48), while all-cause mortality rose by 37% (HR: 1.37, 95% CI: 1.19–1.58). These findings suggest that cobalt ion exposure may represent an underrecognized cardiovascular risk factor.

While local tissue reactions, such as aseptic lymphocyte-dominated vasculitis-associated lesions, have been extensively studied (28–31), the systemic effects of chronic low-dose cobalt ion concentration exposure resulting from metal objects remain inadequately characterized. Given that cobalt ions enter systemic circulation, its potential hematologic (e.g., anemia) and cardiovascular effects warrant further investigation. Therefore, this study systematically evaluates the association between blood cobalt ion concentrations, anemia, and CVD using data from the 2015–2018 National Health and Nutrition Examination Survey (NHANES). Additionally, we explore potential dose–response relationships to address existing research gaps and provide scientific evidence for a better understanding of the systemic health implications of cobalt ion exposure.

## Methods

### Data source and population

The NHANES is an independent cross-sectional study conducted by the National Center for Health Statistics of the Centers for Disease Control and Prevention, designed to assess the health and nutritional status of adults and children in the U.S. A unique feature of this survey is its combination of interviews and physical examinations. The NHANES interviews included demographic, socioeconomic, dietary, and health-related questions. The examination component consists of medical, dental, and physiological measurements, as well as laboratory tests. This study utilized data from two NHANES cycles, 2015–2016 and 2017–2018, which received approval from the NCHS Ethics Review Board with informed consent secured from all participants.

**Figure 1:**
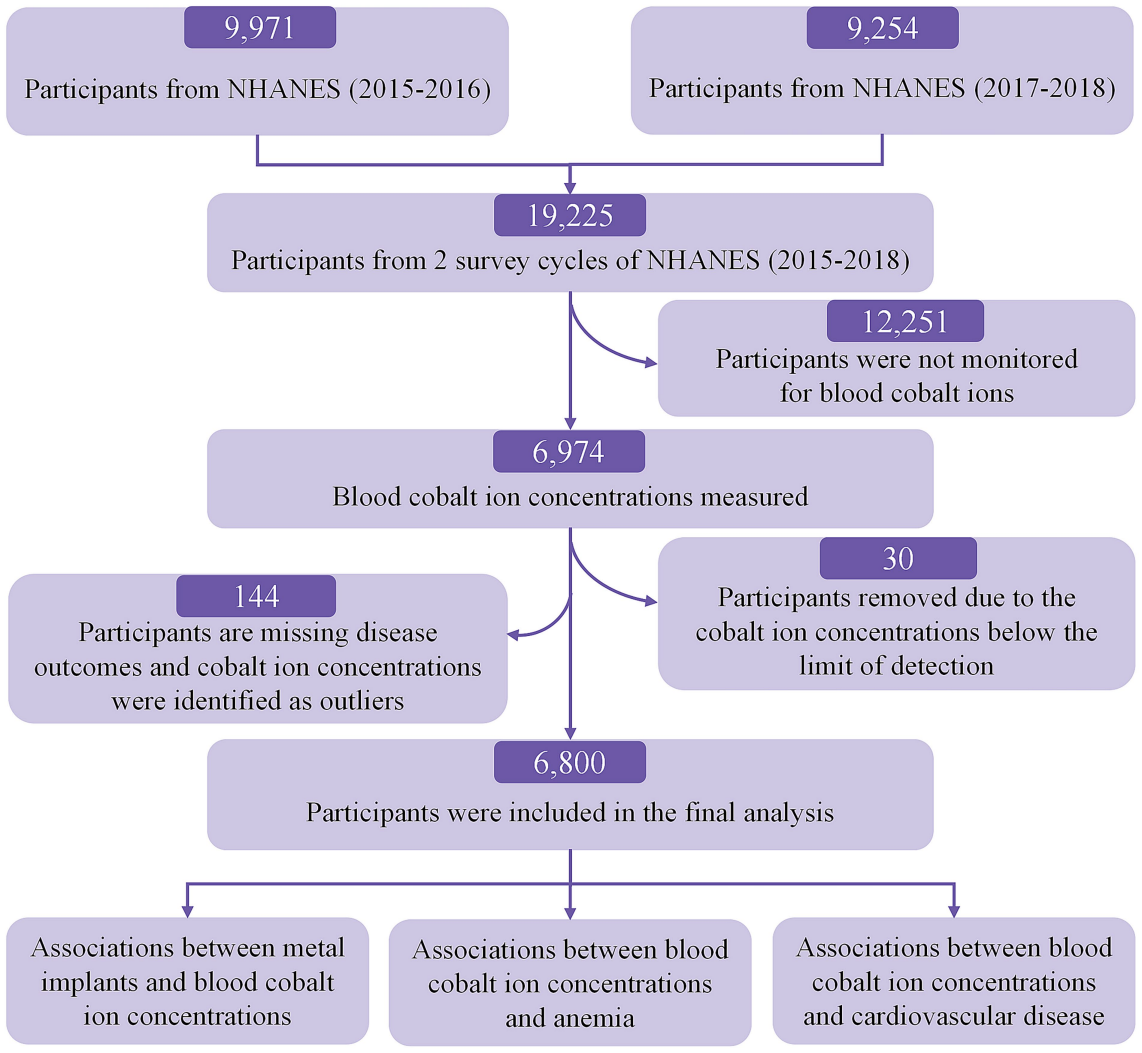
Flowchart shows the study participant selection.

The screening flowchart is shown in Figure 1. Initially, 19,225 participants were enrolled in the 2015–2018 NHANES cycles. Participants were excluded if they lacked blood cobalt ion measurements (*N* = 12,251). Additionally, we excluded 30 participants with blood cobalt ion concentrations below the limit of detection, 70 participants with missing data on CVD and anemia, and 74 participants with extreme outliers in blood cobalt ion concentrations.

### Blood cobalt ion concentrations

Blood cobalt ion concentrations were measured only in participants 40 years of age and older. Whole blood concentrations of cobalt were precisely quantified using inductively coupled plasma mass spectrometry. Comprehensive guidelines for specimen collection and handling are detailed in the Supplementary methods and available on the NHANES website ([2015–2016 cycle]^1^) and ([2017–2018 cycle]^2^). The lower limit of detection for cobalt was 1.02 nmol/L. To explore potential non-linear relationships, the study categorized participants into three groups based on their blood cobalt ion concentrations, focusing on the connection between these levels and designated diseases.

### Cardiovascular disease and anemia

CVD consisted of angina pectoris, arrhythmia, heart attack, heart failure, myocardial infarction, and stroke. Participants who answered “Yes” to the research question “Ever told you had congestive angina pectoris/arrhythmia/heart attack/heart failure/myocardial infarction/stroke?” were classified as having the corresponding disease. Anemia was recognized through anti-anemia medication history, self-reports, or Hb concentrations (males <13.0 g/dL; females <12.0 g/dL) (32, 33).

### Covariates

This study meticulously gathered health outcome data for all participants, categorizing each data type for clarity. Hypertension was identified by self-reports, a history of antihypertensive medication use, or having a systolic pressure exceeding 140 mmHg and/or a diastolic pressure above 90 mmHg (34). Diabetes was defined by self-reported diagnosis, an HbA1c level of 6.5% or higher, or a history of using diabetes medications (34). Alcohol consumption was classified as more than two daily drinks for men and more than one for women. Smoking status was categorized as current, former, or never based on a threshold of 100 cigarettes (35). Comprehensive data for this study are accessible on the NHANES website.^3^

### Statistical analysis

Continuous variables are reported as the mean ± standard deviation (SD), while categorical variables are presented as numbers and proportions. We used logistic regression models for analyzing the relationships between cobalt ion concentrations and anemia and cardiovascular diseases. The chi-square test was applied to categorical data, the Kruskal–Wallis test was used for quantitative data, and the Wald test was used for regression analysis. Our multivariate models adjusted for factors such as sex, age, race, marital status, education level, poverty level index, smoking, alcohol drinking, physical activity, hypertension, diabetes, and total cholesterol. To ascertain the link between cobalt ion concentrations and diseases, we calculated regression coefficients and 95% confidence intervals (CIs) using both unadjusted and adjusted regression analyses. For covariates with missing data, we created five imputed datasets and applied multiple imputation using chained equations with a Markov Chain Monte Carlo method, subsequently pooling the results. Sensitivity analyses were conducted by comparing the multiple imputations with analyses excluding participants with missing covariate data.

Additionally, we performed stratified analyses based on demographic characteristics and the underlying disease to explore potential interactions. To assess the dose–response relationship between blood cobalt ion concentrations and disease outcomes, we used a restricted cubic spline (RCS) function with three knots set at the 10th, 50th, and 90th percentiles. Complemented by threshold effect analysis to discern any significant deviations between the linear and 2-fold piecewise models. A 2-fold piecewise model was applied to pinpoint the optimal inflection point for blood cobalt ion concentrations under the assumption of significant disparities. Additionally, a log-likelihood ratio test was conducted to compare the one linear model with the 2-fold piecewise model. A two-sided *p*-value of <0.05 was considered to indicate statistical significance. Statistical analyses were performed using R version 4.4.2 (R Foundation for Statistical Computing) and the Free Statistics analysis platform (version 2.1.1, Beijing, China).

## Results

### Characteristics

A total of 6,800 participants (3,283 males and 3,517 females) were rigorously selected for the final analysis. These participants were classified into three groups based on their blood cobalt ion concentrations, as detailed in Table 1. The mean age of participants was 60.17 years. Individuals with higher blood cobalt ion levels were more likely to be female, non-drinkers, physically inactive, and diagnosed with hypertension, anemia, or cardiovascular disease (excluding myocardial infarction). They were also more likely to have metal object implants. Additionally, they exhibited lower BMI, waist circumference, and total cholesterol levels. Supplementary Table S1 presents the baseline characteristics of participants after excluding those with missing covariate data. The distributions were highly consistent between the original dataset and multiple imputations, except for education level and alcohol consumption, where minor differences were observed. Furthermore, Supplementary Figure S1 illustrates the distribution of imputed data following multiple imputation. The results indicate that after five iterations of interpolation, the imputed data demonstrated a high degree of consistency with the original dataset, with no detected outliers or significant deviations. This suggests that the multiple imputation approach used in this study provides a robust and reliable dataset for subsequent analyses.

**Table 1:** Baseline characteristics of participants stratified by blood cobalt ion concentrations.

Variables	Total (*N* = 6,800)	Q1 (*N* = 2,621)	Q2 (*N* = 2,187)	Q3 (*N* = 1992)	*p*-value
Age (y)	60.17 ± 11.95	58.57 ± 11.20	60.97 ± 11.62	61.40 ± 12.98	**<0.001**
Cobalt (nmol/L)	2.98 ± 1.69	1.94 ± 0.24	2.60 ± 0.18	4.79 ± 2.19	**<0.001**
BMI (kg/m^2^)	29.98 ± 6.91	30.33 ± 6.78	29.88 ± 6.81	29.62 ± 7.15	**<0.01**
Waist (cm)	102.76 ± 15.86	104.15 ± 15.48	102.39 ± 15.79	101.35 ± 16.29	**<0.001**
Total cholesterol (mg/dL)	188.16 ± 41.51	191.40 ± 42.00	187.10 ± 40.81	185.06 ± 41.35	**<0.001**
Sex					**<0.001**
Male	3,283 (48.28)	1,591 (60.70)	988 (45.18)	704 (35.34)	
Female	3,517 (51.72)	1,030 (39.30)	1,199 (54.82)	1,288 (64.66)	
Race					**<0.001**
Mexican American	1,005 (14.78)	392 (14.96)	337 (15.41)	276 (13.86)	
Other Hispanic	795 (11.69)	359 (13.70)	246 (11.25)	190 (9.54)	
Non-Hispanic White	2,398 (35.26)	876 (33.42)	753 (34.43)	769 (38.60)	
Non-Hispanic Black	1,504 (22.12)	637 (24.30)	435 (19.89)	432 (21.69)	
Other race	1,098 (16.15)	357 (13.62)	416 (19.02)	325 (16.32)	
Marital status					**<0.001**
Married	3,861 (56.78)	1,560 (59.52)	1,218 (55.69)	1,083 (54.37)	
Widowed	737 (10.84)	192 (7.33)	261 (11.93)	284 (14.26)	
Divorced	980 (14.41)	370 (14.12)	312 (14.27)	298 (14.96)	
Separated	267 (3.93)	99 (3.78)	99 (4.53)	69 (3.46)	
Never married	585 (8.60)	241 (9.19)	183 (8.37)	161 (8.08)	
Living with partner	370 (5.44)	159 (6.07)	114 (5.21)	97 (4.87)	
Education level					**0.04**
Less than 9th grade	843 (12.40)	315 (12.02)	303 (13.85)	225 (11.30)	
9–11th Grade	810 (11.91)	329 (12.55)	239 (10.93)	242 (12.15)	
High school graduate	1,535 (22.57)	609 (23.24)	502 (22.95)	424 (21.29)	
Some college or AA degree	1,997 (29.37)	754 (28.77)	654 (29.90)	589 (29.57)	
College graduate or above	1,615 (23.75)	614 (23.43)	489 (22.36)	512 (25.70)	
Poverty level index					0.11
≤1.30	2,219 (32.63)	865 (33.00)	707 (32.33)	647 (32.48)	
1.31–1.85	1,109 (16.31)	402 (15.34)	346 (15.82)	361 (18.12)	
>1.85	3,472 (51.06)	1,354 (51.66)	1,134 (51.85)	984 (49.40)	
Smoking					0.49
Non-users	3,723 (54.75)	1,403 (53.53)	1,220 (55.78)	1,100 (55.22)	
Current smoking	1,156 (17.00)	455 (17.36)	356 (16.28)	345 (17.32)	
Past smoking	1,921 (28.25)	763 (29.11)	611 (27.94)	547 (27.46)	
Alcohol drinking					**0.01**
No	4,015 (59.04)	1,490 (56.85)	1,328 (60.72)	1,197 (60.09)	
Yes	2,785 (40.96)	1,131 (43.15)	859 (39.28)	795 (39.91)	
Physical activity					**0.02**
No	3,930 (57.79)	1,483 (56.58)	1,245 (56.93)	1,202 (60.34)	
Yes	2,870 (42.21)	1,138 (43.42)	942 (43.07)	790 (39.66)	
Hypertension					**<0.01**
No	3,164 (46.53)	1,283 (48.95)	966 (44.17)	915 (45.93)	
Yes	3,636 (53.47)	1,338 (51.05)	1,221 (55.83)	1,077 (54.07)	
Diabetes					0.64
No	4,871 (71.63)	1,878 (71.65)	1,580 (72.25)	1,413 (70.93)	
Yes	1,929 (28.37)	743 (28.35)	607 (27.75)	579 (29.07)	
Anemia					**<0.001**
No	6,227 (91.57)	2,522 (96.22)	2,051 (93.78)	1,654 (83.03)	
Yes	573 (8.43)	99 (3.78)	136 (6.22)	338 (16.97)	
Cardiovascular disease					**<0.001**
No	5,540 (81.47)	2,235 (85.27)	1,791 (81.89)	1,514 (76.00)	
Yes	1,260 (18.53)	386 (14.73)	396 (18.11)	478 (24.00)	
Angina pectoris					**<0.001**
No	6,509 (95.72)	2,535 (96.72)	2,092 (95.66)	1,882 (94.48)	
Yes	291 (4.28)	86 (3.28)	95 (4.34)	110 (5.52)	
Arrhythmia					**<0.001**
No	6,545 (96.25)	2,559 (97.63)	2,106 (96.30)	1,880 (94.38)	
Yes	255 (3.75)	62 (2.37)	81 (3.70)	112 (5.62)	
Heart attack					**<0.001**
No	6,351 (93.40)	2,477 (94.51)	2,052 (93.83)	1,822 (91.47)	
Yes	449 (6.60)	144 (5.49)	135 (6.17)	170 (8.53)	
Heart failure					**<0.001**
No	6,411 (94.28)	2,501 (95.42)	2,085 (95.34)	1,825 (91.62)	
Yes	389 (5.72)	120 (4.58)	102 (4.66)	167 (8.38)	
Myocardial infarction					0.29
No	6,554 (96.38)	2,533 (96.64)	2,112 (96.57)	1,909 (95.83)	
Yes	246 (3.62)	88 (3.36)	75 (3.43)	83 (4.17)	
Stroke					**<0.001**
No	6,368 (93.65)	2,499 (95.35)	2,044 (93.46)	1,825 (91.62)	
Yes	432 (6.35)	122 (4.65)	143 (6.54)	167 (8.38)	
Metal					**<0.001**
No	4,961 (72.96)	2,065 (78.79)	1,605 (73.39)	1,291 (64.81)	
Yes	1,839 (27.04)	556 (21.21)	582 (26.61)	701 (35.19)	

y, year; kg, kilogram, cm, centimeter; Q, quartile; SD, standard deviation; BMI, body mass index; N, number. Values are mean ± SD or *n* (%). *p* < 0.05 means significant statistical differences. Bold values indicate a statistically significant result (*p* < 0.05).

### Association between metal implants and blood cobalt ion concentrations

Supplementary Table S2 presents the baseline characteristics of participants stratified by the presence or absence of metal implants. Individuals with implants were more likely to be older, male, and have a greater burden of comorbidities. Additionally, they exhibited higher blood cobalt ion concentrations. To further explore the association between metal implants and blood cobalt ion concentrations, we conducted a multivariable linear regression analysis (Supplementary Table S3). The results demonstrated a positive association across all models (crude model: *β* = 0.41, 95% CI: 0.32–0.50; model 1: *β* = 0.42, 95% CI: 0.33–0.51; model 2: *β* = 0.43, 95% CI: 0.33–0.52; model 3: *β* = 0.42, 95% CI: 0.33–0.51). Similarly, in sex-stratified analyses, the association remained significant in Model 3 for both males (*β* = 0.43, 95% CI: 0.32–0.53) and females (*β* = 0.42, 95% CI: 0.27–0.56). These findings indicate that individuals with metal implants have significantly higher blood cobalt ion concentrations compared to those without implants, reinforcing the potential systemic impact of implant-related cobalt exposure.

### Association between blood cobalt ion concentrations and anemia

**Figure 2:**
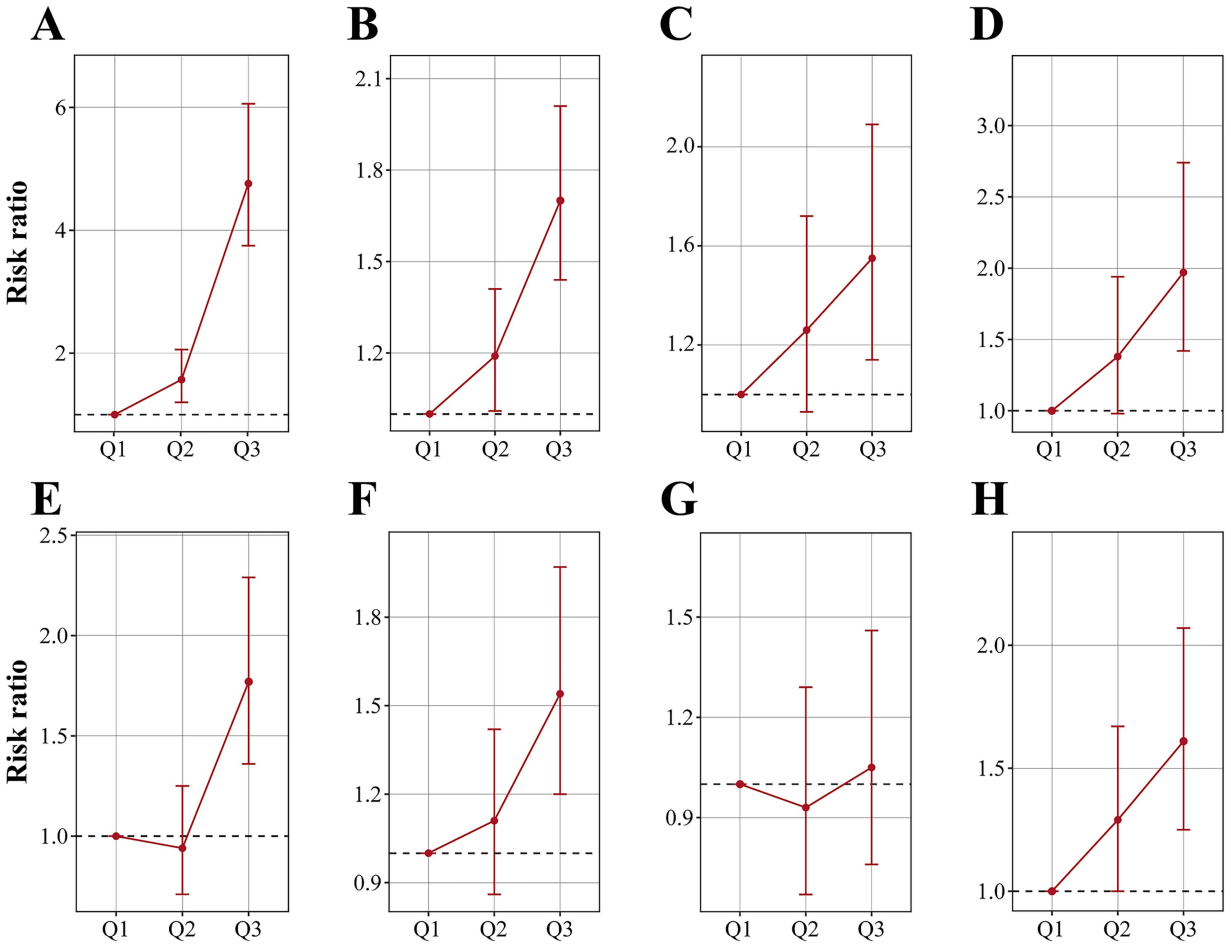
Risk ratios and 95% confidence intervals of anemia **(A)**, CVD **(B)**, angina pectoris **(C)**, arrhythmia **(D)**, heart attack **(E)**, heart failure **(F)**, myocardial infarction **(G)** and stroke **(H)** by tripartite of blood cobalt ion concentrations. The analysis was adjusted by age, sex, race, marital status, education level, poverty level index, smoking, alcohol drinking, physical activity, body mass index, waist, hypertension, diabetes, and total cholesterol.

**Figure 3:**
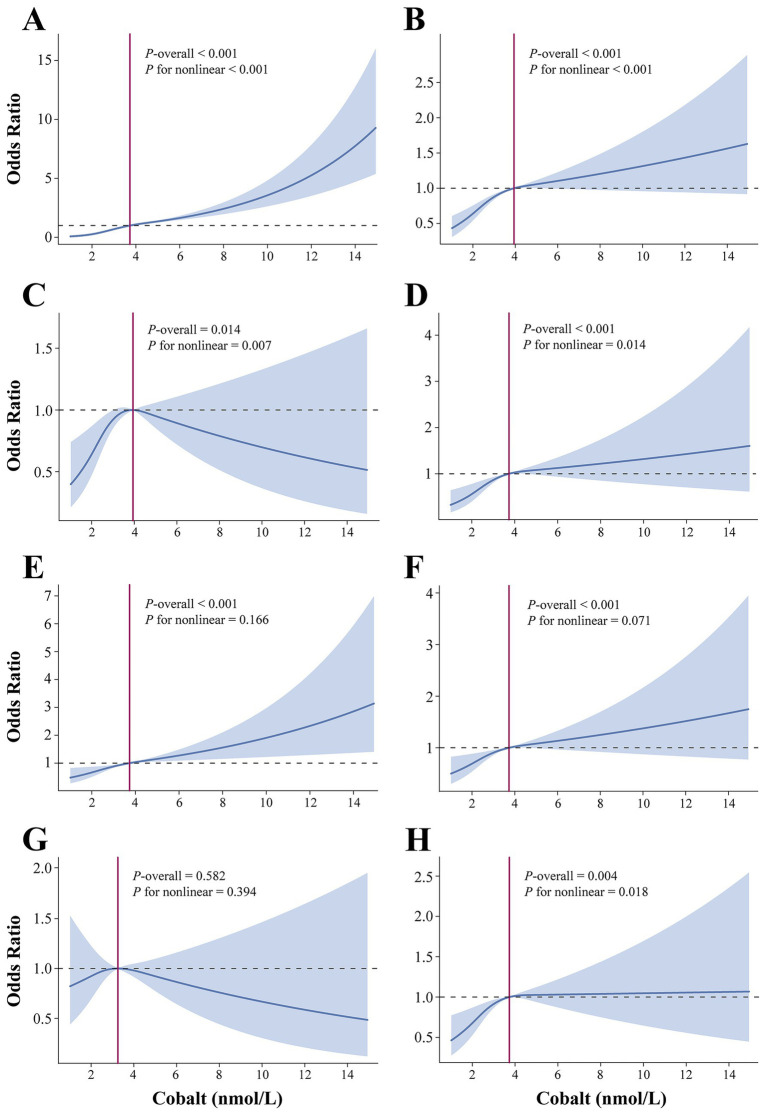
Dose–responsive relationship of the blood cobalt ion concentrations and anemia **(A)**, CVD **(B)**, angina pectoris **(C)**, arrhythmia **(D)**, heart attack **(E)**, heart failure **(F)**, myocardial infarction **(G)**, and stroke **(H)**. The analysis was adjusted by age, sex, race, marital status, education level, poverty level index, smoking, alcohol drinking, physical activity, body mass index, waist, hypertension, diabetes, and total cholesterol.

Table 2 summarizes the results of the multivariate regression analysis. When cobalt ion concentrations were treated as a continuous variable, a significant positive association with anemia was observed across all models. In Model 3, there was a 36% higher risk of anemia for every 1 nmol/L rise in blood cobalt ion levels (OR: 1.36; 95% CI: 1.31–1.41). After conversion to categorical variables, the Q3 group had a markedly elevated risk of anemia compared to the Q1 group (OR: 4.76; 95% CI: 3.75–6.06) (Figure 2A). Furthermore, the results from the RCS analysis demonstrated a non-linear positive association between cobalt ion concentrations and anemia after full adjustment for covariates (Figure 3A). To further validate this non-linear relationship, a threshold effect analysis was performed. However, no significant inflection point was identified, suggesting that the observed non-linearity was primarily driven by the magnitude of OR differences before and after the estimated threshold (Table 3).

**Table 2:** Multivariate logistic regression analysis for the effect of blood cobalt ion concentrations on anemia and cardiovascular disease.

Variables	Crude model		Model 1		Model 2		Model 3	
	OR (95% CI)	*p*-value	OR (95% CI)	*p*-value	OR (95% CI)	*p*-value	OR (95% CI)	*p*-value
Anemia								
Cobalt	1.35 (1.30, 1.40)	**<0.001**	1.34 (1.30, 1.39)	**<0.001**	1.35 (1.30, 1.40)	**<0.001**	1.36 (1.31, 1.41)	**<0.001**
Q1	Ref.		Ref.		Ref.		Ref.	
Q2	1.69 (1.30, 2.20)	**<0.001**	1.58 (1.21, 2.06)	**<0.001**	1.59 (1.22, 2.08)	**<0.001**	1.57 (1.20, 2.06)	**<0.001**
Q3	5.21 (4.13, 6.57)	**<0.001**	4.75 (3.74, 6.02)	**<0.001**	4.85 (3.82, 6.16)	**<0.001**	4.76 (3.75, 6.06)	**<0.001**
*P* for trend		**<0.001**		**<0.001**		**<0.001**		**<0.001**
Cardiovascular disease								
Cobalt	1.10 (1.06, 1.13)	**<0.001**	1.10 (1.06, 1.14)	**<0.001**	1.11 (1.07, 1.15)	**<0.001**	1.11 (1.07, 1.15)	**<0.001**
Q1	Ref.		Ref.		Ref.		Ref.	
Q2	1.28 (1.10, 1.49)	**0.002**	1.18 (1.00, 1.38)	**0.05**	1.21 (1.03, 1.42)	**0.02**	1.19 (1.01, 1.41)	**0.04**
Q3	1.83 (1.57, 2.12)	**<0.001**	1.67 (1.42, 1.95)	**<0.001**	1.75 (1.48, 2.06)	**<0.001**	1.70 (1.44, 2.01)	**<0.001**
*P* for trend		**<0.001**		**<0.001**		**<0.001**		**<0.001**
Angina pectoris								
Cobalt	1.05 (0.98, 1.11)	0.16	1.03 (0.97, 1.10)	0.34	1.03 (0.97, 1.11)	0.33	1.04 (0.97, 1.11)	0.29
Q1	Ref.		Ref.		Ref.		Ref.	
Q2	1.34 (0.99, 1.80)	0.05	1.25 (0.92, 1.69)	0.16	1.27 (0.94, 1.72)	0.12	1.26 (0.93, 1.72)	0.13
Q3	1.72 (1.29, 2.30)	**<0.001**	1.54 (1.14, 2.08)	**0.004**	1.57 (1.17, 2.13)	**0.003**	1.55 (1.14, 2.09)	**0.005**
*P* for trend		**<0.001**		**0.004**		**0.003**		**0.005**
Arrhythmia								
Cobalt	1.14 (1.08, 1.20)	**<0.001**	1.12 (1.06, 1.19)	**<0.001**	1.12 (1.05, 1.19)	**<0.001**	1.12 (1.05, 1.19)	**<0.001**
Q1	Ref.		Ref.		Ref.		Ref.	
Q2	1.59 (1.13, 2.22)	**0.01**	1.37 (0.97, 1.93)	0.07	1.40 (0.99, 1.97)	**0.05**	1.38 (0.98, 1.94)	0.07
Q3	2.46 (1.79, 3.37)	**<0.001**	1.99 (1.43, 2.75)	**<0.001**	2.01 (1.45, 2.79)	**<0.001**	1.97 (1.42, 2.74)	**<0.001**
*P* for trend		**<0.001**		**<0.001**		**<0.001**		**<0.001**
Heart attack								
Cobalt	1.09 (1.04, 1.14)	**<0.001**	1.10 (1.04, 1.15)	**<0.001**	1.10 (1.04, 1.16)	**<0.001**	1.10 (1.04, 1.16)	**<0.001**
Q1	Ref.		Ref.		Ref.		Ref.	
Q2	1.13 (0.89, 1.44)	0.32	1.11 (0.86, 1.42)	0.42	1.12 (0.87, 1.44)	0.37	1.11 (0.86, 1.42)	0.43
Q3	1.60 (1.28, 2.02)	**<0.001**	1.54 (1.21, 1.96)	**<0.001**	1.59 (1.24, 2.03)	**<0.001**	1.54 (1.20, 1.97)	**<0.001**
*P* for trend		**<0.001**		**<0.001**		**<0.001**		**<0.001**
Heart failure								
Cobalt	1.13 (1.08, 1.19)	**<0.001**	1.13 (1.08, 1.19)	**<0.001**	1.14 (1.08, 1.20)	**<0.001**	1.14 (1.09, 1.21)	**<0.001**
Q1	Ref.		Ref.		Ref.		Ref.	
Q2	1.02 (0.78, 1.34)	0.89	0.94 (0.71, 1.24)	0.66	0.95 (0.72, 1.26)	0.73	0.94 (0.71, 1.25)	0.67
Q3	1.91 (1.50, 2.43)	**<0.001**	1.70 (1.32, 2.19)	**<0.001**	1.81 (1.40, 2.35)	**<0.001**	1.77 (1.36, 2.29)	**<0.001**
*P* for trend		**<0.001**		**<0.001**		**<0.001**		**<0.001**
Myocardial infarction								
Cobalt	1 (0.93, 1.08)	0.95	0.97 (0.90, 1.06)	0.53	0.97 (0.90, 1.06)	0.53	0.97 (0.89, 1.06)	0.52
Q1	Ref.		Ref.		Ref.		Ref.	
Q2	1.02 (0.75, 1.40)	0.89	0.95 (0.69, 1.31)	0.76	0.95 (0.69, 1.31)	0.75	0.93 (0.67, 1.29)	0.68
Q3	1.25 (0.92, 1.70)	0.15	1.09 (0.79, 1.50)	0.61	1.10 (0.80, 1.51)	0.56	1.05 (0.76, 1.46)	0.75
*P* for trend		0.12		0.52		0.47		0.64
Stroke								
Cobalt	1.08 (1.03, 1.13)	**0.003**	1.06 (1.01, 1.12)	**0.02**	1.07 (1.01, 1.13)	**0.02**	1.07 (1.01, 1.13)	**0.02**
Q1	Ref.		Ref.		Ref.		Ref.	
Q2	1.43 (1.12, 1.84)	**0.005**	1.27 (0.99, 1.64)	0.06	1.31 (1.01, 1.69)	**0.04**	1.29 (1.00, 1.67)	0.05
Q3	1.87 (1.47, 2.39)	**<0.001**	1.60 (1.25, 2.05)	**<0.001**	1.65 (1.28, 2.12)	**<0.001**	1.61 (1.25, 2.07)	**<0.001**
*P* for trend		**<0.001**		**<0.001**		**<0.001**		**<0.001**

OR, odds ratio; Q, quartile; CI, confidence interval. Values are OR (95% CI). *p* < 0.05 means significant statistical differences.

Crude model: unadjusted.

Model 1 adjust for age, sex, and race.

Model 2 adjust for: age, sex, race, marital status, education level, poverty level index, smoking, alcohol drinking, physical activity, body mass index, and waist.

Model 3 adjust for: age, sex, race, marital status, education level, poverty level index, smoking, alcohol drinking, physical activity, body mass index, waist, hypertension, diabetes, and total cholesterol. Bold values indicate a statistically significant result (*p* < 0.05).

**Table 3:** Threshold effect analysis of blood cobalt ion concentrations on anemia and cardiovascular disease.

Variables	Anemia	Cardiovascular disease	Angina pectoris	Arrhythmia
	OR (95% CI) *p*-value	OR (95% CI) *p*-value	OR (95% CI) *p*-value	OR (95% CI) *p*-value
One linear model	1.36 (1.31, 1.41) < 0.001	1.11 (1.07, 1.15) < 0.001	1.04 (0.97, 1.11) 0.29	1.12 (1.05, 1.19) < 0.001
2-fold piecewise model				
Inflection point (nmol/L)	3.73	3.94	3.94	3.73
Cobalt < inflection point	1.95 (1.56, 2.44) < 0.001	1.27 (1.12, 1.45) < 0.001	1.35 (1.08, 1.70) 0.009	1.35 (1.01, 1.82) 0.043
Cobalt ≥ inflection point	1.15 (1.08, 1.22) < 0.001	0.97 (0.89, 1.04) 0.359	0.92 (0.78, 1.07) 0.266	1.00 (0.90, 1.12) 0.998
*P* for Log-likelihood ratio	<0.001	<0.001	0.015	0.015

Variables	Heart attack	Heart failure	Myocardial infarction	Stroke
One linear model	1.10 (1.04, 1.16) < 0.001	1.14 (1.09, 1.21) < 0.001	0.97 (0.89, 1.06) 0.521	1.07 (1.01, 1.13) 0.016
2-fold piecewise model				
Inflection point (nmol/L)	3.73	3.73	3.25	3.73
Cobalt < inflection point	1.23 (0.99, 1.54) 0.064	1.17 (0.91, 1.49) 0.218	1.13 (0.81, 1.58) 0.472	1.29 (1.03, 1.61) 0.025
Cobalt ≥ inflection point	1.03 (0.93, 1.12) 0.601	1.02 (0.93, 1.12) 0.635	0.93 (0.81, 1.07) 0.324	0.97 (0.87, 1.07) 0.495
*P* for Log-likelihood ratio	0.101	0.013	0.611	0.022

OR, odds ratio; CI, confidence interval.

### Association between blood cobalt ion concentrations and cardiovascular disease

In Model 3, cobalt ion concentrations demonstrated a positive association with CVD, regardless of whether it was treated as a continuous or categorical variable (Table 2). When considered as a continuous variable, each 1 nmol/L increase in blood cobalt ion concentrations was associated with an 11% higher risk of CVD (OR: 1.11; 95% CI: 1.07–1.15). When converted to categorical variables, the Q3 group had a significantly increased risk of CVD compared to Q1 (OR: 1.70; 95% CI: 1.44–2.01) (Figure 2B). The RCS analysis revealed a non-linear association between cobalt ion concentrations and CVD in model 3 (Figure 3B). Further threshold effect analysis identified an inflection point at 3.94 nmol/L. Below this inflection point, cobalt ion concentrations were significantly associated with increased CVD risk (OR: 1.27; 95% CI: 1.12–1.45), whereas no significant association was observed beyond this inflection point (Table 3).

### Association between cobalt and subtypes of cardiovascular diseases

Among the six CVD subtypes, when cobalt ion concentrations were treated as a continuous variable, Model 3 demonstrated a positive association between cobalt ion concentrations and arrhythmia (OR: 1.04; 95% CI: 0.97–1.11), heart attack (OR: 1.10; 95% CI: 1.04–1.16), heart failure (OR: 1.14; 95% CI: 1.09–1.21), and stroke (OR: 1.07; 95% CI: 1.01–1.13). However, no significant association was found with angina pectoris (OR: 1.04; 95% CI: 0.97–1.11) or myocardial infarction (OR: 0.97; 95% CI: 0.89–1.06) (Table 2). When cobalt ion concentrations were analyzed as a categorical variable, myocardial infarction remained unassociated with cobalt ion exposure (Figures 2C–H). The summarized results further confirmed the absence of an association between cobalt ions and myocardial infarction (Figure 3G; Table 3). For the remaining five CVD subtypes, RCS and threshold effect analyses revealed non-linear associations between cobalt ion and angina pectoris, arrhythmia, and stroke, with inflection points at 3.94 nmol/L, 3.73 nmol/L, and 3.73 nmol/L, respectively. Below these thresholds, cobalt ion was significantly associated with increased disease risk, whereas the association disappeared beyond the inflection points (Figures 3C,D,H; Table 3). Conversely, the threshold effect validation was not significant for heart attack and heart failure, indicating a linear positive association between cobalt ion concentrations and these conditions (Figures 3E,F; Table 3).

### Stratified analysis

**Figure 4:**
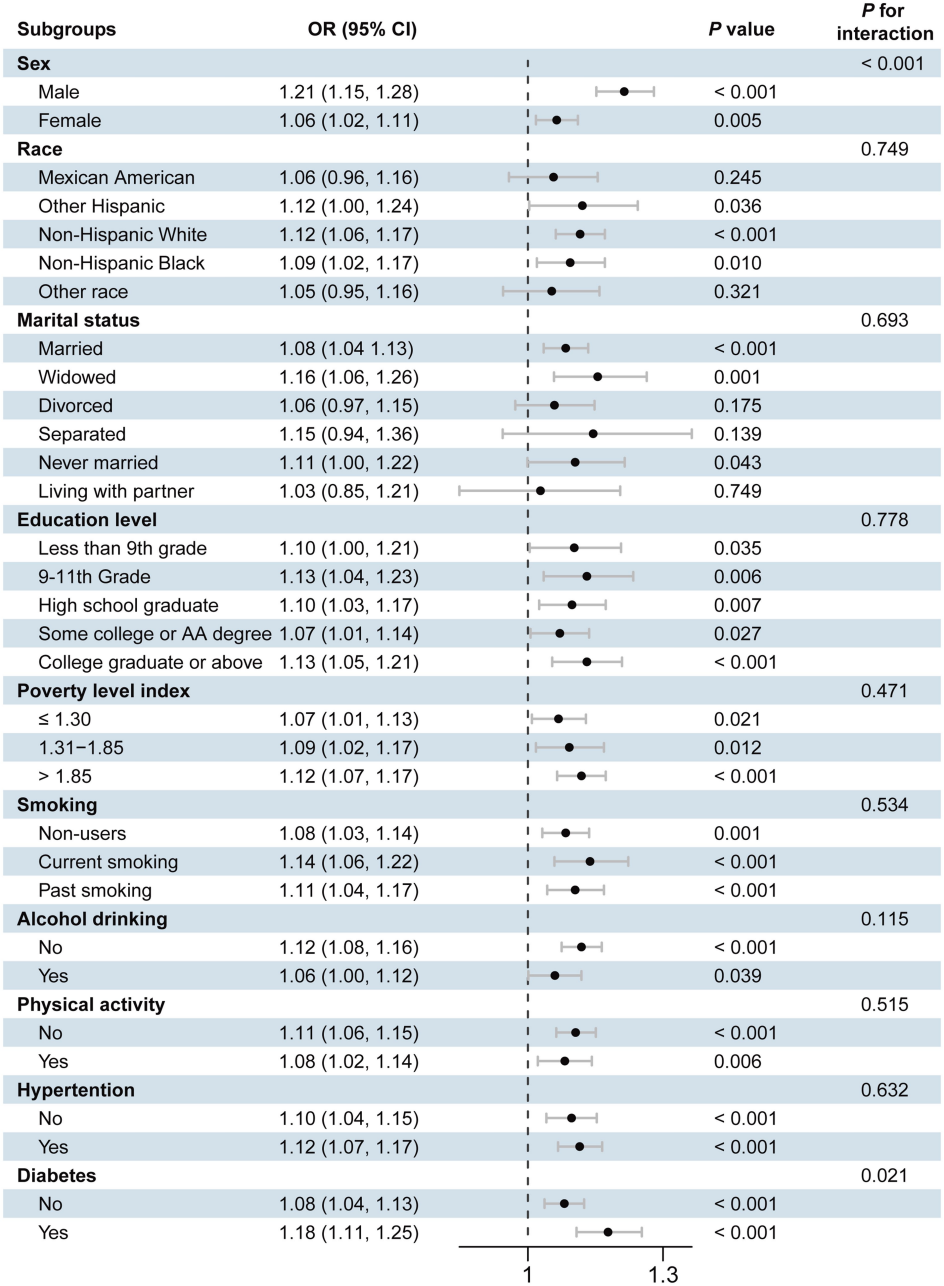
Forest plot of stratified analysis of the association of blood cobalt ion concentrations with cardiovascular disease. The analysis was adjusted by age, sex, race, marital status, education level, poverty level index, smoking, alcohol drinking, physical activity, body mass index, waist, hypertension, diabetes, and total cholesterol.

To further evaluate the robustness of the associations between cobalt ion, anemia, and CVD, we conducted stratified analyses based on demographic characteristics and comorbidities. As shown in Supplementary Figure S2, the positive association between cobalt ion concentrations and anemia remained statistically significant across all subgroups (*p* < 0.05). For cobalt ion and CVD, subgroup analyses revealed variability in associations across race and marital status, although the overall results remained stable (Figure 4). Among CVD subtypes, the harmful effects of cobalt ion on angina pectoris, arrhythmia, heart attack, heart failure, and stroke were more pronounced in males. Engaging in physical activity mitigated the adverse effects of cobalt ion on heart attack and heart failure, while maintaining normal blood pressure appeared to counteract the detrimental impact of cobalt ion on arrhythmia and heart attack. Additionally, normal blood glucose levels were associated with a reduced effect of cobalt ion on stroke (Supplementary Figures S3–S7). Notably, no significant association was observed between cobalt ion and myocardial infarction across all subgroups, consistent with the previous findings (Supplementary Figure S8).

Interaction analyses revealed that marital status modified the association between cobalt ion concentrations and anemia, with individuals living with a partner exhibiting a higher risk of anemia. For CVD (Figure 4), significant interactions were observed between sex and diabetes status, with higher disease risk in men and individuals with diabetes. In arrhythmia (Supplementary Figure S4) and heart failure (Supplementary Figure S6), only sex exhibited an interaction effect, with men demonstrating a greater risk. No significant interaction effects were observed for angina pectoris (Supplementary Figure S3), heart attack (Supplementary Figure S5), stroke (Supplementary Figure S7), or myocardial infarction (Supplementary Figure S8).

## Discussion

Our study provides novel evidence on the systemic toxicity of cobalt ions released from metal-containing objects, revealing robust associations between elevated blood cobalt ion concentrations and increased risks of anemia and CVDs in a large population-based cohort. Multiple linear regression analyses showed that blood cobalt ion levels were 0.42 nmol/L higher in participants with metallic objects compared with those without, confirming that the implantation of metal objects is a significant source of cobalt ion exposure. Notably, each 1 nmol/L increase in blood cobalt ion concentrations was associated with a 36% higher risk of anemia. Strikingly, non-linear associations were identified for CVD and its subtypes (angina pectoris, arrhythmia, and stroke), with threshold effects suggesting toxicity manifests predominantly below 3.94 nmol/L, whereas heart attack and heart failure demonstrated linear risk escalation with cobalt ion concentrations. The absence of association with myocardial infarction highlights potential mechanistic distinctions in cobalt-induced cardiovascular pathology. Subgroup analyses further emphasized heightened susceptibility in males and modifiable protective effects of physical activity and normal blood pressure, underscoring the interplay between cobalt ion toxicity and host factors. These findings align with experimental evidence of cobalt-induced oxidative stress and erythropoietic suppression, yet extend prior clinical observations by comprehensively delineating population-level risks across CVD subtypes and quantifying exposure thresholds relevant to prosthetic surveillance.

Although the core focus of this study is on cobalt exposure from metal implants, it is necessary to fully consider other potential sources when interpreting the results in order to more comprehensively understand the causes of elevated blood cobalt ion concentrations in different populations. Dietary intake represents an important source of cobalt exposure in the body. Studies have shown that in mining areas of the Democratic Republic of Congo, diet is the primary source of cobalt exposure, especially through drinking water, vegetables, and fruits. In these regions, urinary cobalt concentrations in adults and children were 4.5 and 6.6 times higher, respectively, than in control areas (36). Additionally, occupational exposure is an important factor in affecting cobalt ion levels in the body. While respiratory inhalation has traditionally been considered the primary absorption route, multiple studies of Swedish cemented carbide factory workers provide strong quantitative evidence that dermal contact is also an important pathway that cannot be overlooked. Even with adequate respiratory protection in place, dermal exposure alone can lead to changes in blood cobalt ion concentrations (37, 38), suggesting that occupational health interventions need to cover more exposure routes.

Orthopedic implant cobalt ion toxicity, a form of progeria linked to poisoning, remains incompletely understood, potentially impacting millions of high-risk individuals (39). Notably, as a mitochondrial toxin, cobalt ions especially affect organs with high metabolic activity, such as the brain and heart (40, 41). The first case of cobalt ion poisoning resulting from modern primary THA was reported in 2010 (42, 43), followed by additional reports in 2011 and 2014 (44, 45). Despite these reports, research on metal implant-associated cobalt ion toxicity remains limited, making it essential to investigate its potential health consequences. Unravelling the variations in ion concentrations necessitates an understanding of the release mechanisms involved in MoM THA (46). There are primarily two release mechanisms. First, the normal wear of the prosthesis releases small, uniform metal particles, chiefly chromium. However, when the prosthesis is improperly placed, there is an accelerated release, predominantly of larger cobalt particles (47). The second mechanism involves mechanically assisted crevice corrosion at the joint, leading to the release of both cobalt and chromium but predominantly cobalt. Our study further supports this; participants with metal implants had significantly higher blood cobalt ion concentrations (*p* < 0.001).

In the last century, cobalt was used in the treatment of anemia with the intention of utilizing its ability to induce tissue hypoxia, thereby stimulating the activity of heme oxidase and promoting the production of red blood cells (48, 49). However, this therapeutic application is contradicted by cobalt’s structural mimicry of iron ions. As a divalent metal cation, the cobalt ion competes with the iron ion for binding to transferrin and ferroportin, disrupting systemic iron homeostasis—a fundamental mechanism in anemia pathogenesis (50, 51). In addition to this, cobalt ions inhibit the activity of 5-aminolevulinic acid synthase (ALA synthase), which reduces the production of 5-aminolevulinic acid and thus inhibits heme synthesis (52). Cobalt ion also reduces heme availability by inducing heme oxygenase activity and accelerating heme degradation (53, 54). These mechanisms work together to ultimately lead to anemia. Our study corroborates this mechanistic evidence by demonstrating a significant positive correlation between cobalt exposure and anemia risk (OR: 1.36; 95% CI: 1.31–1.41).

The toxicity risks posed by metal implants in the body, particularly to the heart, are often underestimated (55). Our study included six CVD subtypes, of which five exhibited a positive association with cobalt ions. The cardiotoxic effects of cobalt ions are primarily mediated through a combination of oxidative stress, mitochondrial dysfunction, and calcium homeostasis disruption. Cobalt exposure significantly increases reactive oxygen species levels in cardiomyocytes, inducing oxidative stress that damages cell membranes, proteins, and DNA (56–58). Simultaneously, cobalt ions inhibit mitochondrial respiratory chain enzyme activity, reducing ATP production, leading to energy metabolism dysfunction and further exacerbating oxidative stress (59, 60). Additionally, cobalt ions interfere with calcium ion channels, causing intracellular calcium overload, which results in excessive myocardial contraction and functional impairment (61). The lack of association between cobalt ion and myocardial infarction is mechanistically consistent with these findings. While cobalt ion exacerbates oxidative stress and mitochondrial dysfunction, it does not directly promote atherosclerotic plaque rupture, which is the hallmark of myocardial infarction. This explains why cobalt ion is linked to other CVDs but not myocardial infarction. Such divergence underscores the specificity of cobalt ion-induced cardiovascular toxicity, with a greater impact on myocardial electrical and structural damage rather than coronary thrombotic events.

Sex-specific susceptibility, particularly the greater vulnerability observed in men, is likely attributed to sex differences in cardiac electrophysiology and oxidative stress responses. Evidence suggests that men have weaker antioxidant defense mechanisms in the heart, making them more susceptible to cobalt-induced oxidative damage. Additionally, higher testosterone levels in men have been shown to modulate cardiac electrophysiology by inhibiting the RISK/SAFE pathway and regulating potassium channel expression, further increasing myocardial susceptibility to cobalt toxicity (58, 62). Conversely, the protective effect of physical activity may be mediated through the regulation of redox balance and endothelial function improvement. Kumral et al. (63) demonstrated that regular exercise mitigates cardiac and endothelial dysfunction as well as oxidative stress-related damage. Moreover, physical activity promotes the upregulation of antioxidant enzymes, thereby reducing oxidative stress and preserving vascular function (64).

These mechanisms align with our findings, bridging population-level observations with molecular pathophysiology while underscoring cobalt ion as a systemic toxicant with organ-specific vulnerabilities. Future research should validate these pathways in human tissues and explore the interactions between cobalt ion exposure and genetic or environmental cofactors to refine risk stratification strategies.

Currently, there are no definitive guidelines for diagnosing cobalt toxicity associated with metal implants, but the literature has proposed several diagnostic criteria: (1) metal implant-induced elevated blood cobalt ion concentrations, (2) at least two test results consistent with cobalt poisoning, and (3) exclusion of other causes (65). Magnetic resonance imaging (MRI) effectively detects pseudotumors following MoM THA (66). Cardiac MRI provides valuable insights into cardiomyopathy resulting from cobalt ion toxicity (67). The final diagnosis should be made by integrating pathological, hematological, imaging, and surgical outcomes.

This study offers new epidemiological insights into how blood cobalt ion impacts American health. We reinforced the validity of these associations across different diseases using diverse models, bolstering the credibility of our findings. However, it is crucial to acknowledge certain limitations. First, the cross-sectional nature of this study hinders our ability to infer a causal link between cobalt exposure and disease development. Second, the reliance on questionnaire surveys for diagnosing CVD might lead to recall bias. Third, this study did not account for dietary and environmental exposure, which are primary sources of cobalt. However, our findings also indicate that metal implants contribute to elevated blood cobalt ion concentrations, highlighting the need for future research to consider dietary influences on health outcomes. Fourth, while we adjusted for numerous confounders, the potential influence of other known or unknown risk factors cannot be entirely dismissed.

## Conclusion

Our study provides compelling evidence that prosthesis implantation leads to significantly elevated blood cobalt ion concentrations, which are associated with the increased risks of anemia and CVDs. Subtype analyses further validated significant associations between cobalt exposure and angina pectoris, arrhythmia, heart attack, heart failure, and stroke, whereas no relationship was observed with myocardial infarction. Notably, cobalt ion exhibited nonlinear associations with angina pectoris, arrhythmia, and stroke. This study is among the first to establish a threshold effect of cobalt ion toxicity on CVD risk, offering novel insights into its dose–response relationship. Given the widespread use of metal implants, further longitudinal research is essential to elucidate the underlying mechanisms, identify at-risk populations, and develop clinical monitoring strategies to mitigate potential health hazards.

## Data Availability

Publicly available datasets were analyzed in this study. All data generated or analyzed during this study are included in this published article and the NHANES website (https://www.cdc.gov/nchs/nhanes/index.htm).

## Footnotes

^1^ https://wwwn.cdc.gov/Nchs/Nhanes/2015-2016/CRCO_I.htm

^2^ https://wwwn.cdc.gov/Nchs/Nhanes/2017-2018/CRCO_J.htm

^3^ https://www.cdc.gov/nchs/nhanes/index.htm
