# Supplementary material for: Association between blood cobalt ion concentrations and anemia and cardiovascular diseases: novel evidence of toxicity resulting from metal implants
Source: Front Nutr. 2025 Sep 3;12:1614771. doi: 10.3389/fnut.2025.1614771 (PMC12441040; doi:10.3389/fnut.2025.1614771)
Supplement: Supplementary file 1 [file Data_Sheet_1.pdf]

Supplemental Online Content

Supplementary Methods

Supplementary Table 1. Baseline characteristics of participants stratified by blood cobalt ion concentrations after excluding missing values of covariates.

Supplementary Table 2. Baseline characteristics of participants stratified by the presence of metal implants.

Supplementary Table 3. Multivariate linear regression analysis for effect of metal implants on blood cobalt ion concentrations.

Supplementary Figure 1. Multiple imputation strip plot of continuous variables body mass index, waist circumference and total cholesterol.

Supplementary Figure 2. Forest plot of stratified analysis of the association of blood cobalt ion concentrations on anemia.

Supplementary Figure 3. Forest plot of stratified analysis of the association of blood cobalt ion concentrations on angina pectoris.

Supplementary Figure 4. Forest plot of stratified analysis of the association of blood cobalt ion concentrations on arrhythmia.

Supplementary Figure 5. Forest plot of stratified analysis of the association of blood cobalt ion concentrations on heart attack.

Supplementary Figure 6. Forest plot of stratified analysis of the association of blood cobalt ion concentrations on heart failure.

Supplementary Figure 7. Forest plot of stratified analysis of the association of blood cobalt ion concentrations on stroke.

Supplementary Figure 8. Forest plot of stratified analysis of the association of blood cobalt ion concentrations on myocardial infarction.

Supplementary Methods

Measurement of blood Co ion concentrations

The method involves converting the sample into aerosol, which is then heated to high temperatures (6000–8000 °K) in a plasma, atomizing and ionizing the sample. The ions enter a mass spectrometer where they pass through ion optics and a collision cell (QCell™) with helium, reducing argon-based polyatomic interferences. The ions then reach a detector, generating electrical signals that are converted into digital information that is used to indicate the intensity of the ions. The intensity of ions detected while aspirating an unknown sample is translated into an elemental concentration through comparison of the analyte to internal standard signal ratio of the unknown with the ratio obtained when aspirating calibration standards.

Supplementary Table 1. Baseline characteristics of participants stratified by blood cobalt ion concentrations after excluding missing values of covariates.

Variable	Total (N = 3000)	Q1 (N = 1000)	Q2 (N = 1089)	Q3 (N = 911)	*P* value
**Age (y)**	58.06 ± 11.36	56.16 ± 10.48	58.36 ± 11.12	59.80 ± 12.23	**< 0.001**
**Cobalt (nmol/L)**	2.86 ± 1.61	1.83 ± 0.20	2.43 ± 0.19	4.51 ± 2.08	**< 0.001**
**BMI (kg/m^2)**	29.95 ± 6.69	30.19 ± 6.25	29.89 ± 6.82	29.76 ± 6.99	**0.04**
**Waist (cm)**	102.74 ± 15.61	104.03 ± 14.77	102.45 ± 15.82	101.66 ± 16.18	**< 0.01**
**Total cholesterol (mg/dL)**	190.30 ± 42.29	194.79 ± 42.47	188.50 ± 40.31	187.52 ± 44.00	**< 0.001**
**Sex**					**< 0.001**
Male	1599 (53.30)	664 (66.40)	591 (54.27)	344 (37.76)	
Female	1401 (46.70)	336 (33.60)	498 (45.73)	567 (62.24)	
**Race**					**< 0.01**
Mexican American	408 (13.60)	142 (14.20)	142 (13.04)	124 (13.61)	
Other Hispanic	344 (11.47)	131 (13.10)	128 (11.75)	85 (9.33)	
Non-Hispanic White	1213 (40.43)	377 (37.70)	444 (40.77)	392 (43.03)	
Non-Hispanic Black	630 (21.00)	237 (23.70)	214 (19.65)	179 (19.65)	
Other race	405 (13.50)	113 (11.30)	161 (14.78)	131 (14.38)	
**Marital status**					**< 0.001**
Married	1763 (58.77)	618 (61.80)	639 (58.68)	506 (55.54)	
Widowed	219 (7.30)	48 (4.80)	80 (7.35)	91 (9.99)	
Divorced	463 (15.43)	141 (14.10)	159 (14.60)	163 (17.89)	
Separated	109 (3.63)	38 (3.80)	40 (3.67)	31 (3.40)	
Never married	249 (8.30)	92 (9.20)	95 (8.72)	62 (6.81)	
Living with partner	197 (6.57)	63 (6.30)	76 (6.98)	58 (6.37)	
**Education level**					0.41
Less than 9th grade	206 (6.87)	73 (7.30)	78 (7.16)	55 (6.04)	
9-11th Grade	315 (10.50)	109 (10.90)	118 (10.84)	88 (9.66)	

High school graduate	673 (22.43)	232 (23.20)	254 (23.32)	187 (20.53)	
Some college or AA degree	958 (31.93)	316 (31.60)	344 (31.59)	298 (32.71)	
College graduate or above	848 (28.27)	270 (27.00)	295 (27.09)	283 (31.06)	
**Poverty level index**					0.44
≤ 1.30	790 (26.33)	270 (27.00)	293 (26.91)	227 (24.92)	
1.31-1.85	444 (14.80)	133 (13.30)	168 (15.43)	143 (15.70)	
> 1.85	1766 (58.87)	597 (59.70)	628 (57.67)	541 (59.39)	
**Smoking**					0.24
Non-users	1476 (49.20)	476 (47.60)	528 (48.48)	472 (51.81)	
Current smoking	612 (20.40)	222 (22.20)	222 (20.39)	168 (18.44)	
Past smoking	912 (30.40)	302 (30.20)	339 (31.13)	271 (29.75)	
**Alcohol drinking**					0.23
No	1739 (57.97)	559 (55.90)	649 (59.60)	531 (58.29)	
Yes	1261 (42.03)	441 (44.10)	440 (40.40)	380 (41.71)	
**Physical activity**					0.21
No	1553 (51.77)	533 (53.30)	541 (49.68)	479 (52.58)	
Yes	1447 (48.23)	467 (46.70)	548 (50.32)	432 (47.42)	
**Hypertension**					**0.05**
No	1525 (50.83)	537 (53.70)	549 (50.41)	439 (48.19)	
Yes	1475 (49.17)	463 (46.30)	540 (49.59)	472 (51.81)	
**Diabetes**					0.60
No	2269 (75.63)	746 (74.60)	833 (76.49)	690 (75.74)	
Yes	731 (24.37)	254 (25.40)	256 (23.51)	221 (24.26)	
**Anemia**					**< 0.001**
No	2812 (93.73)	975 (97.50)	1041 (95.59)	796 (87.38)	
Yes	188 (6.27)	25 (2.50)	48 (4.41)	115 (12.62)	
**Cardiovascular disease**					**< 0.001**

No	2538 (84.60)	888 (88.80)	928 (85.22)	722 (79.25)	
Yes	462 (15.40)	112 (11.20)	161 (14.78)	189 (20.75)	
**Angina pectoris**					**0.03**
No	2895 (96.50)	976 (97.60)	1050 (96.42)	869 (95.39)	
Yes	105 (3.50)	24 (2.40)	39 (3.58)	42 (4.61)	
**Arrhythmia**					**< 0.001**
No	2917 (97.23)	986 (98.60)	1059 (97.25)	872 (95.72)	
Yes	83 (2.77)	14 (1.40)	30 (2.75)	39 (4.28)	
**Heart attack**					**< 0.01**
No	2828 (94.27)	958 (95.80)	1031 (94.67)	839 (92.10)	
Yes	172 (5.73)	42 (4.20)	58 (5.33)	72 (7.90)	
**Heart failure**					**< 0.001**
No	2871 (95.70)	970 (97.00)	1051 (96.51)	850 (93.30)	
Yes	129 (4.30)	30 (3.00)	38 (3.49)	61 (6.70)	
**Myocardial infarction**					0.30
No	2920 (97.30)	973 (97.30)	1060 (97.34)	887 (97.37)	
Yes	80 (2.70)	27 (2.70)	29 (2.66)	24 (2.63)	
**Stroke**					**< 0.001**
No	2860 (95.33)	973 (97.30)	1034 (94.95)	853 (93.63)	
Yes	140 (4.67)	27 (2.70)	55 (5.05)	58 (6.37)	
**Metal**					**< 0.001**
No	2200 (73.33)	789 (78.90)	814 (74.75)	597 (65.53)	
Yes	800 (26.67)	211 (21.10)	275 (25.25)	314 (34.47)	

Abbreviations: BMI, body mass index; N, number. Values are mean ± SD or n (%). $P < 0.05$ means significant statistical differences.

Supplementary Table 2. Baseline characteristics of participants stratified by the presence of metal implants.

Variable	Total (N = 6800)	No (N = 4961)	Yes (N = 1839)	*P* value
**Age (y)**	60.17 ± 11.95	58.49 ± 11.60	64.70 ± 11.69	**< 0.001**
**Cobalt (nmol/L)**	2.98 ± 1.69	2.87 ± 1.59	3.28 ± 1.89	**< 0.001**
**BMI (kg/m^2)**	29.98 ± 6.91	29.88 ± 6.99	30.25 ± 6.67	0.05
**Waist (cm)**	102.76 ± 15.86	102.02 ± 15.87	104.76 ± 15.68	**< 0.001**
**Total cholesterol (mg/dL)**	188.16 ± 41.51	189.58 ± 41.87	184.34 ± 40.30	**< 0.001**
**Sex**				**0.03**
Male	3283 (48.28)	2355 (47.47)	928 (50.46)	
Female	3517 (51.72)	2606 (52.53)	911 (49.54)	
**Race**				**< 0.001**
Mexican American	1005 (14.78)	776 (15.64)	229 (12.45)	
Other Hispanic	795 (11.69)	598 (12.05)	197 (10.71)	
Non-Hispanic White	2398 (35.26)	1528 (30.80)	870 (47.31)	
Non-Hispanic Black	1504 (22.12)	1178 (23.75)	326 (17.73)	
Other race	1098 (16.15)	881 (17.76)	217 (11.80)	
**Marital**				**< 0.001**
Married	3861 (56.78)	2832 (57.09)	1029 (55.95)	
Widowed	737 (10.84)	488 (9.84)	249 (13.54)	
Divorced	980 (14.41)	712 (14.35)	268 (14.57)	
Separated	267 (3.93)	207 (4.17)	60 (3.26)	
Never married	585 (8.60)	445 (8.97)	140 (7.61)	
Living with partner	370 (5.44)	277 (5.58)	93 (5.06)	
**Education**				**< 0.001**
Less than 9th grade	843 (12.40)	653 (13.16)	190 (10.33)	
9-11th Grade	810 (11.91)	609 (12.28)	201 (10.93)	
High school graduate	1535 (22.57)	1098 (22.13)	437 (23.76)	
Some college or AA degree	1997 (29.37)	1395 (28.12)	602 (32.74)	
College graduate or above	1615 (23.75)	1206 (24.31)	409 (22.24)	
**Poverty level index**				**< 0.001**
≤ 1.30	2219 (32.63)	1686 (33.99)	533 (28.98)	
1.31-1.85	1109 (16.31)	774 (15.60)	335 (18.22)	
> 1.85	3472 (51.06)	2501 (50.41)	971 (52.80)	
**Smoke**				**< 0.001**
Non-users	3723 (54.75)	2840 (57.25)	883 (48.02)	
Current smoking	1156 (17.00)	856 (17.25)	300 (16.31)	
Past smoking	1921 (28.25)	1265 (25.50)	656 (35.67)	
**Drink**				**< 0.001**
No	4015 (59.04)	2848 (57.41)	1167 (63.46)	
Yes	2785 (40.96)	2113 (42.59)	672 (36.54)	
**Physical activity**				0.14
No	3930 (57.79)	2840 (57.25)	1090 (59.27)	
Yes	2870 (42.21)	2121 (42.75)	749 (40.73)	
**Hypertension**				**< 0.001**
No	3164 (46.53)	2469 (49.77)	695 (37.79)	
Yes	3636 (53.47)	2492 (50.23)	1144 (62.21)	
**Diabetes**				**< 0.001**
No	4871 (71.63)	3649 (73.55)	1222 (66.45)	
Yes	1929 (28.37)	1312 (26.45)	617 (33.55)	
**Anemia**				**< 0.001**
No	6227 (91.57)	4586 (92.44)	1641 (89.23)	
Yes	573 (8.43)	375 (7.56)	198 (10.77)	
**Cardiovascular disease**				**< 0.001**
No	5540 (81.47)	4328 (87.24)	1212 (65.91)	
Yes	1260 (18.53)	633 (12.76)	627 (34.09)	
**Angina pectoris**				**< 0.001**
No	6509 (95.72)	4831 (97.38)	1678 (91.25)	
Yes	291 (4.28)	130 (2.62)	161 (8.75)	

**Arrhythmia**				**< 0.001**
No	6545 (96.25)	4823 (97.22)	1722 (93.64)	
Yes	255 (3.75)	138 (2.78)	117 (6.36)	
**Heart attack**				**< 0.001**
No	6351 (93.40)	4806 (96.88)	1545 (84.01)	
Yes	449 (6.60)	155 (3.12)	294 (15.99)	
**Heart failure**				**< 0.001**
No	6411 (94.28)	4812 (97.00)	1599 (86.95)	
Yes	389 (5.72)	149 (3.00)	240 (13.05)	
**Myocardial infarction**				**< 0.001**
No	6554 (96.38)	4861 (97.98)	1693 (92.06)	
Yes	246 (3.62)	100 (2.02)	146 (7.94)	
**Stroke**				**< 0.001**
No	6368 (93.65)	4732 (95.38)	1636 (88.96)	
Yes	432 (6.35)	229 (4.62)	203 (11.04)	

Abbreviations: BMI, body mass index; N, number. Values are mean ± SD or n (%). $P < 0.05$ means significant statistical differences.

Supplementary Table 3. Multivariate linear regression analysis for effect of metal implants on blood cobalt ion concentrations.

**Variables**	**Crude model**		**Model 1**		**Model 2**		**Model 3**	
	**β (95% CI)**	***P* value**	**β (95% CI)**	***P* value**	**β (95% CI)**	***P* value**	**β (95% CI)**	***P* value**
**All**								
No	Ref.		Ref.		Ref.		Ref.	
Yes	0.41 (0.32, 0.50)	< **0.001**	0.42 (0.33, 0.51)	< **0.001**	0.43 (0.34, 0.52)	< **0.001**	0.42 (0.33, 0.51)	< **0.001**
**Male**								
No	Ref.		Ref.		Ref.		Ref.	
Yes	0.57 (0.47, 0.68)	< **0.001**	0.44 (0.33, 0.54)	< **0.001**	0.43 (0.33, 0.54)	< **0.001**	0.43 (0.32, 0.53)	< **0.001**
**Female**								
No	Ref.		Ref.		Ref.		Ref.	
Yes	0.28 (0.14, 0.42)	< **0.001**	0.41 (0.26, 0.55)	< **0.001**	0.42 (0.28, 0.57)	< **0.001**	0.42 (0.27, 0.56)	< **0.001**

Abbreviations: OR, Odds Ratio; CI, Confidence interval. Values are β (95% CI). $P < 0.05$ means significant statistical differences.

Crude model: unadjusted.

Model 1 adjust for: Age, Sex, Race.

Model 2 adjust for: Age, Sex, Race, Marital Status, Education Level, Poverty Level Index, Smoking, Alcohol Drinking, Physical Activity, Body Mass Index, Waist.

Model 3 adjust for: Age, Sex, Race, Marital Status, Education Level, Poverty Level Index, Smoking, Alcohol Drinking, Physical Activity, Body Mass Index, Waist, Hypertension, Diabetes, Total cholesterol.

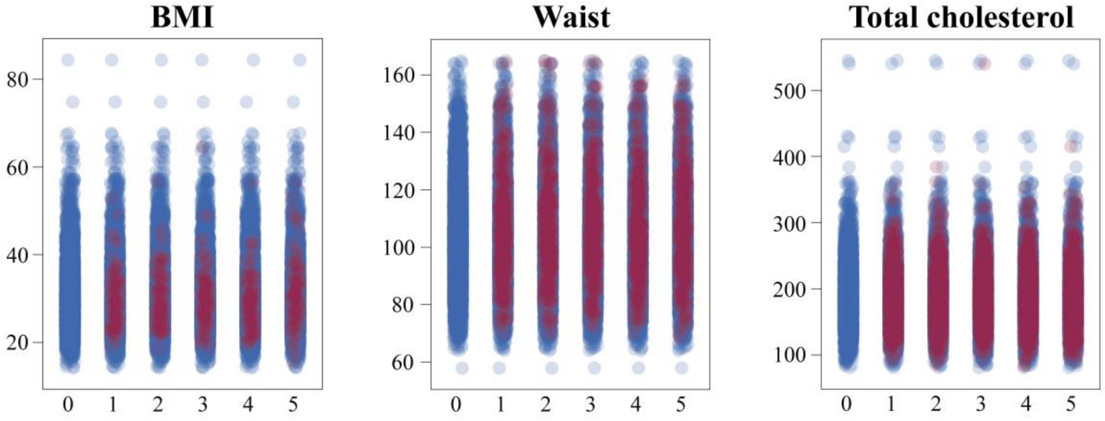

Supplementary Figure 1. Multiple imputation strip plot of continuous variables body mass index, waist circumference and total cholesterol.

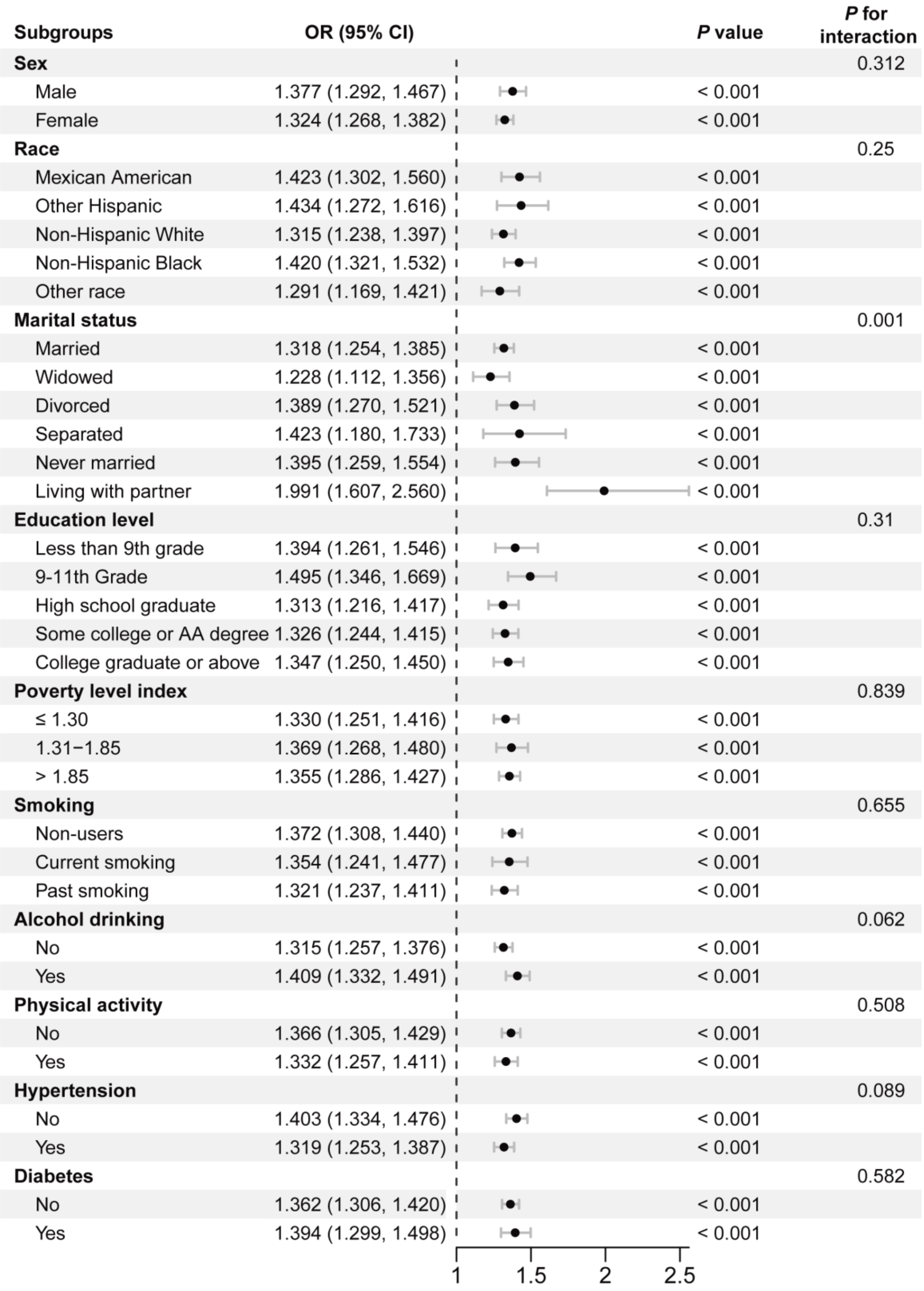

Supplementary Figure 2. Forest plot of stratified analysis of the association of blood cobalt ion concentrations on anemia.

Subgroups	OR (95% CI)	*P* value	*P* for interaction
**Sex**			0.061
Male	1.133 (1.034, 1.230)	0.005	
Female	1.004 (0.909, 1.095)	0.929	
**Race**			0.794
Mexican American	0.990 (0.786, 1.172)	0.923	
Other Hispanic	0.932 (0.674, 1.172)	0.609	
Non-Hispanic White	1.040 (0.947, 1.130)	0.382	
Non-Hispanic Black	1.098 (0.942, 1.244)	0.180	
Other race	1.075 (0.870, 1.257)	0.432	
**Marital status**			0.635
Married	1.019 (0.917, 1.116)	0.700	
Widowed	1.051 (0.902, 1.194)	0.480	
Divorced	1.077 (0.936, 1.213)	0.256	
Separated	1.110 (0.655, 1.503)	0.586	
Never married	0.599 (0.195, 1.100)	0.262	
Living with partner	1.088 (0.758, 1.368)	0.550	
**Education level**			0.733
Less than 9th grade	0.947 (0.726, 1.147)	0.636	
9-11th Grade	1.097 (0.933, 1.251)	0.210	
High school graduate	1.051 (0.907, 1.188)	0.463	
Some college or AA degree	1.015 (0.887, 1.134)	0.815	
College graduate or above	1.096 (0.954, 1.228)	0.149	
**Poverty level index**			0.207
≤ 1.30	0.959 (0.834, 1.077)	0.517	
1.31−1.85	1.047 (0.909, 1.178)	0.478	
> 1.85	1.098 (1.000, 1.191)	0.035	
**Smoking**			0.936
Non-users	1.031 (0.919, 1.137)	0.568	
Current smoking	1.052 (0.917, 1.180)	0.428	
Past smoking	1.059 (0.950, 1.163)	0.263	
**Alcohol drinking**			0.492
No	1.063 (0.980, 1.143)	0.118	
Yes	1.015 (0.902, 1.122)	0.785	
**Physical activity**			0.974
No	1.045(0.965, 1.122)	0.253	
Yes	1.042 (0.920, 1.158)	0.475	
**Hypertension**			0.921
No	1.057 (0.926, 1.176)	0.358	
Yes	1.049 (0.967, 1.129)	0.220	
**Diabetes**			0.656
No	1.041 (0.948, 1.129)	0.363	
Yes	1.072 (0.969, 1.173)	0.150	

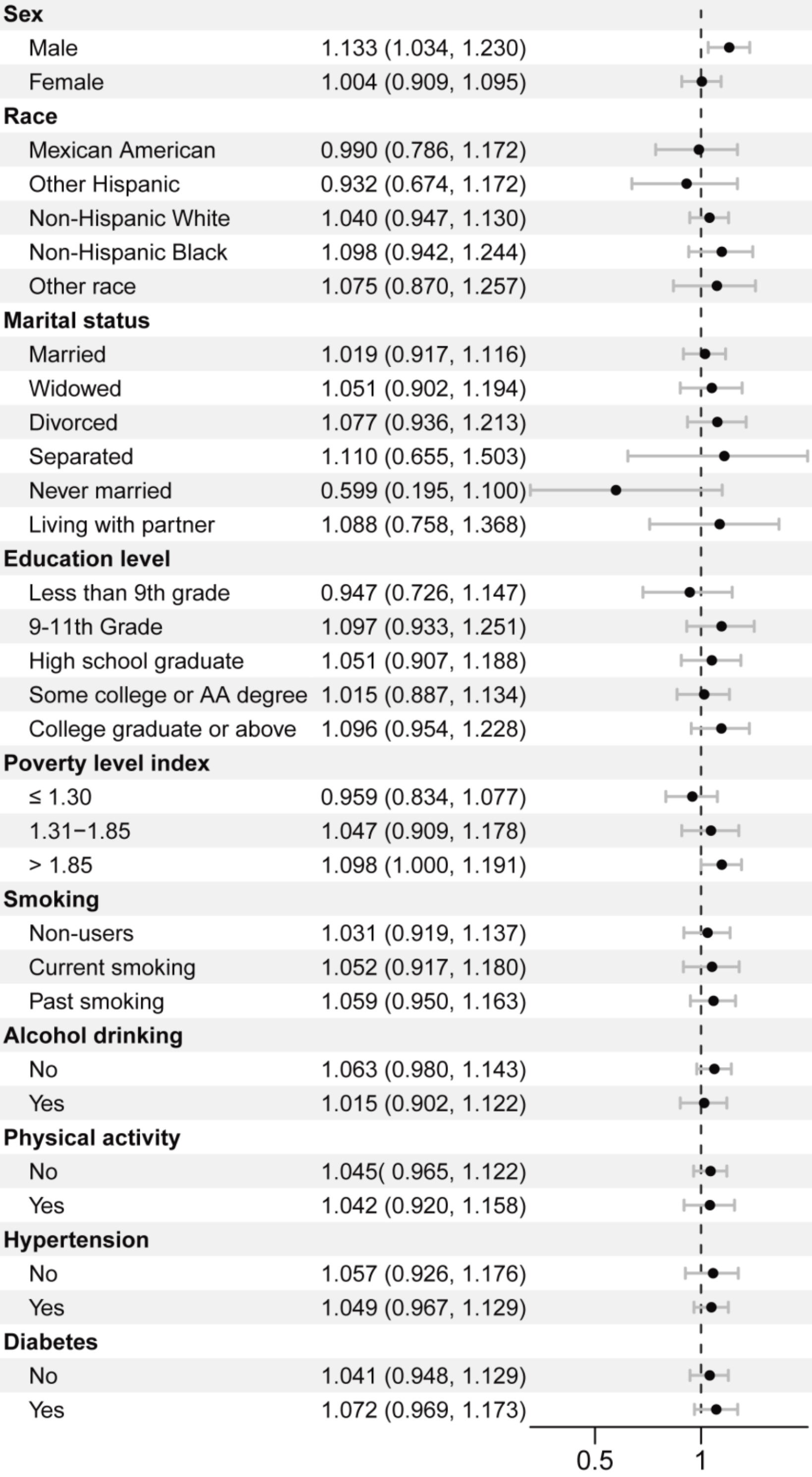

Supplementary Figure 3. Forest plot of stratified analysis of the association of blood cobalt ion concentrations on angina pectoris.

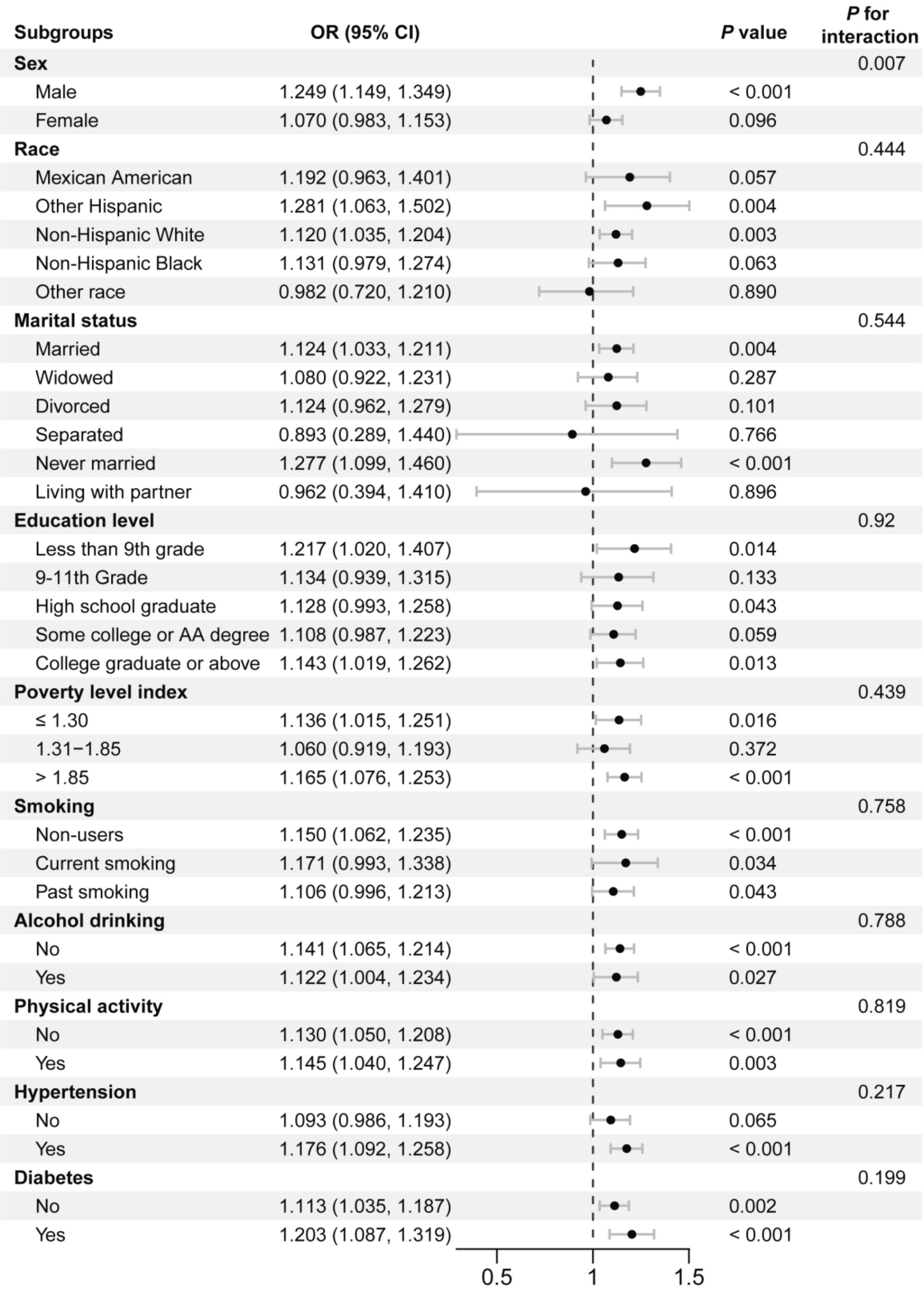

Supplementary Figure 4. Forest plot of stratified analysis of the association of blood cobalt ion concentrations on arrhythmia.

Subgroups	OR (95% CI)	*P* value	*P* for interaction
**Sex**			0.067
Male	1.183 (1.108,1.260)	< 0.001	
Female	1.082 (1.001,1.160)	0.036	
**Race**			0.915
Mexican American	1.072 (0.915, 1.219)	0.332	
Other Hispanic	1.155 (0.993, 1.318)	0.043	
Non-Hispanic White	1.078 (1.003, 1.152)	0.033	
Non-Hispanic Black	1.064 (0.938, 1.185)	0.289	
Other race	1.105 (0.949, 1.251)	0.149	
**Marital status**			0.481
Married	1.092 (1.016, 1.165)	0.011	
Widowed	1.112 (0.986, 1.240)	0.065	
Divorced	1.061 (0.938, 1.180)	0.307	
Separated	1.285 (1.013, 1.580)	0.022	
Never married	0.968 (0.741, 1.163)	0.771	
Living with partner	0.959 (0.645, 1.229)	0.791	
**Education level**			0.971
Less than 9th grade	1.089 (0.947, 1.226)	0.190	
9-11th Grade	1.057 (0.910, 1.197)	0.418	
High school graduate	1.075 (0.962, 1.185)	0.171	
Some college or AA degree	1.103 (1.011, 1.193)	0.020	
College graduate or above	1.115 (0.995, 1.230)	0.042	
**Poverty level index**			0.468
≤ 1.30	1.039 (0.949, 1.127)	0.374	
1.31−1.85	1.101 (1.001, 1.200)	0.036	
> 1.85	1.111 (1.027, 1.193)	0.005	
**Smoking**			0.898
Non-users	1.096 (1.010, 1.178)	0.019	
Current smoking	1.107 (1.000, 1.213)	0.038	
Past smoking	1.076 (0.991, 1.160)	0.065	
**Alcohol drinking**			0.507
No	1.100 (1.037, 1.162)	0.001	
Yes	1.063 (0.971, 1.152)	0.158	
**Physical activity**			0.083
No	1.118 (1.056, 1.180)	< 0.001	
Yes	1.018 (0.920, 1.112)	0.705	
**Hypertension**			0.718
No	1.083 (0.993, 1.168)	0.053	
Yes	1.103 (1.036, 1.170)	0.002	
**Diabetes**			0.854
No	1.101 (1.035, 1.166)	0.002	
Yes	1.091 (1.003, 1.179)	0.034	

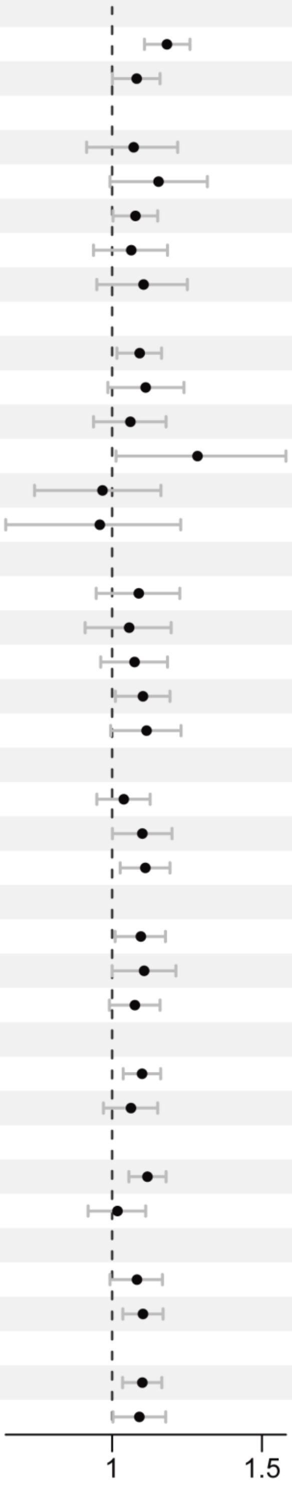

Supplementary Figure 5. Forest plot of stratified analysis of the association of blood cobalt ion concentrations on heart attack.

Subgroups	OR (95% CI)	*P* value	*P* for interaction
**Sex**			0.048
Male	1.221 (1.140,1.303)	< 0.001	
Female	1.110 (1.034,1.184)	0.002	
**Race**			0.93
Mexican American	1.095 (0.924,1.253)	0.232	
Other Hispanic	1.204 (1.026,1.383)	0.014	
Non-Hispanic White	1.124 (1.046,1.202)	< 0.001	
Non-Hispanic Black	1.129 (1.022,1.235)	0.011	
Other race	1.135 (0.950,1.304)	0.109	
**Marital status**			0.147
Married	1.127 (1.048,1.204)	< 0.001	
Widowed	1.115 (0.985,1.244)	0.065	
Divorced	1.041 (0.903,1.173)	0.542	
Separated	1.333 (1.071,1.633)	0.006	
Never married	1.246 (1.091,1.406)	< 0.001	
Living with partner	0.912 (0.568,1.209)	0.621	
**Education level**			0.937
Less than 9th grade	1.129 (0.982,1.272)	0.062	
9-11th Grade	1.177 (1.041,1.314)	0.006	
High school graduate	1.155 (1.051,1.259)	0.002	
Some college or AA degree	1.114 (1.015,1.210)	0.016	
College graduate or above	1.110 (0.961,1.249)	0.111	
**Poverty level index**			0.739
≤ 1.30	1.129 (1.042,1.215)	0.002	
1.31−1.85	1.158 (1.054,1.263)	0.001	
> 1.85	1.104 (1.013,1.191)	0.016	
**Smoking**			0.745
Non-users	1.110 (1.021,1.196)	0.009	
Current smoking	1.165 (1.049,1.280)	0.002	
Past smoking	1.141 (1.057,1.226)	< 0.001	
**Alcohol drinking**			0.914
No	1.132 (1.068,1.196)	< 0.001	
Yes	1.126 (1.032,1.218)	0.005	
**Physical activity**			0.405
No	1.144 (1.082,1.207)	< 0.001	
Yes	1.094 (0.991,1.193)	0.056	
**Hypertension**			0.674
No	1.157 (1.067,1.244)	< 0.001	
Yes	1.133 (1.064,1.202)	< 0.001	
**Diabetes**			0.263
No	1.120 (1.046,1.192)	< 0.001	
Yes	1.185 (1.099,1.273)	< 0.001	

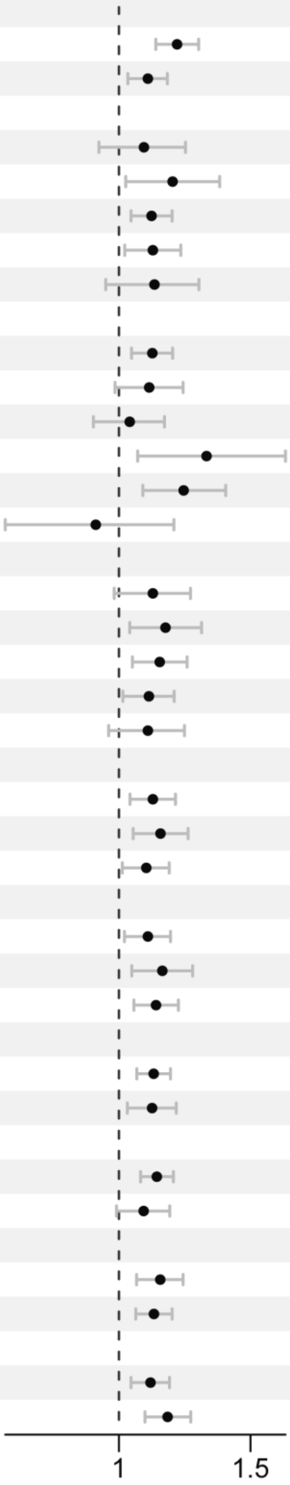

Supplementary Figure 6. Forest plot of stratified analysis of the association of blood cobalt ion concentrations on heart failure.

Subgroups	OR (95% CI)	*P* value	*P* for interaction
**Sex**			0.249
Male	1.123 (1.034, 1.211)	0.004	
Female	1.058 (0.989, 1.125)	0.087	
**Race**			0.789
Mexican American	0.991 (0.802, 1.161)	0.919	
Other Hispanic	1.063 (0.852, 1.263)	0.537	
Non-Hispanic White	1.106 (1.028, 1.183)	0.005	
Non-Hispanic Black	1.052 (0.945, 1.157)	0.318	
Other race	1.086 (0.923, 1.237)	0.262	
**Marital status**			0.756
Married	1.091 (1.013, 1.168)	0.015	
Widowed	1.111 (0.988, 1.235)	0.061	
Divorced	0.989 (0.838, 1.131)	0.887	
Separated	1.046 (0.728, 1.336)	0.758	
Never married	1.003 (0.823, 1.164)	0.975	
Living with partner	1.099 (0.845, 1.331)	0.396	
**Education level**			0.335
Less than 9th grade	0.928 (0.732, 1.108)	0.471	
9-11th Grade	1.112 (0.981, 1.242)	0.074	
High school graduate	1.075 (0.971, 1.177)	0.137	
Some college or AA degree	1.071 (0.972, 1.167)	0.140	
College graduate or above	1.163 (1.033, 1.287)	0.007	
**Poverty level index**			0.314
≤ 1.30	1.042 (0.953, 1.129)	0.338	
1.31−1.85	1.038 (0.927, 1.146)	0.482	
> 1.85	1.124 (1.040, 1.207)	0.002	
**Smoking**			0.601
Non-users	1.047 (0.962, 1.128)	0.260	
Current smoking	1.108 (1.000, 1.215)	0.038	
Past smoking	1.097 (1.005, 1.187)	0.029	
**Alcohol drinking**			0.255
No	1.100 (1.034, 1.165)	0.002	
Yes	1.036 (0.944, 1.124)	0.432	
**Physical activity**			0.502
No	1.064 (1.001, 1.127)	0.038	
Yes	1.105 (1.004, 1.202)	0.029	
**Hypertension**			0.365
No	1.118 (1.027, 1.205)	0.006	
Yes	1.066 (0.997, 1.134)	0.052	
**Diabetes**			0.246
No	1.060 (0.990, 1.128)	0.077	
Yes	1.127 (1.038, 1.217)	0.003	

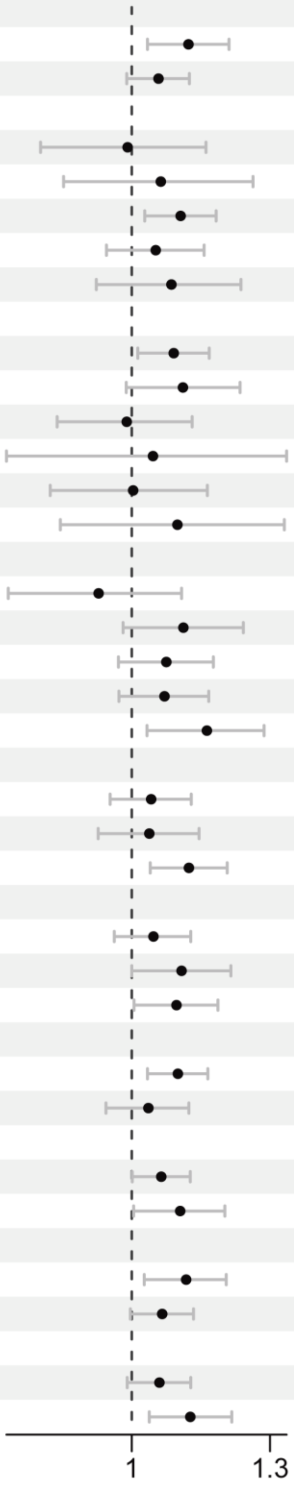

Supplementary Figure 7. Forest plot of stratified analysis of the association of blood cobalt ion concentrations on stroke.

Subgroups	OR (95% CI)	*P* value	*P* for interaction
**Sex**			0.603
Male	1.056 (0.947, 1.159)	0.289	
Female	1.015 (0.899, 1.124)	0.794	
**Race**			0.81
Mexican American	0.885 (0.653, 1.094)	0.346	
Other Hispanic	0.955 (0.695, 1.197)	0.736	
Non-Hispanic White	1.009 (0.897, 1.113)	0.875	
Non-Hispanic Black	0.996 (0.798, 1.176)	0.970	
Other race	1.064 (0.856, 1.249)	0.511	
**Marital status**			0.718
Married	1.008 (0.907, 1.105)	0.866	
Widowed	0.913 (0.714, 1.100)	0.405	
Divorced	0.923 (0.678, 1.142)	0.537	
Separated	1.232 (0.838, 1.606)	0.174	
Never married	0.945 (0.576, 1.241)	0.760	
Living with partner	1.088 (0.651, 1.436)	0.644	
**Education level**			0.481
Less than 9th grade	0.875 (0.642, 1.092)	0.321	
9-11th Grade	1.050 (0.866, 1.219)	0.566	
High school graduate	1.042 (0.873, 1.198)	0.608	
Some college or AA degree	0.915 (0.747, 1.071)	0.331	
College graduate or above	1.061 (0.909, 1.199)	0.398	
**Poverty level index**			0.625
≤ 1.30	0.947 (0.800, 1.084)	0.474	
1.31−1.85	1.048 (0.893, 1.192)	0.516	
> 1.85	1.000 (0.885, 1.107)	0.993	
**Smoking**			0.283
Non-users	1.003 (0.889, 1.109)	0.964	
Current smoking	1.119 (0.933, 1.291)	0.165	
Past smoking	0.939 (0.807, 1.063)	0.366	
**Alcohol drinking**			0.631
No	1.007 (0.916, 1.092)	0.882	
Yes	0.965 (0.811, 1.107)	0.646	
**Physical activity**			0.613
No	0.982 (0.884, 1.074)	0.710	
Yes	1.023 (0.893, 1.144)	0.721	
**Hypertension**			0.064
No	1.088 (0.967, 1.200)	0.122	
Yes	0.940 (0.837, 1.040)	0.262	
**Diabetes**			0.496
No	1.028 (0.923, 1.126)	0.584	
Yes	0.974 (0.853, 1.090)	0.669	

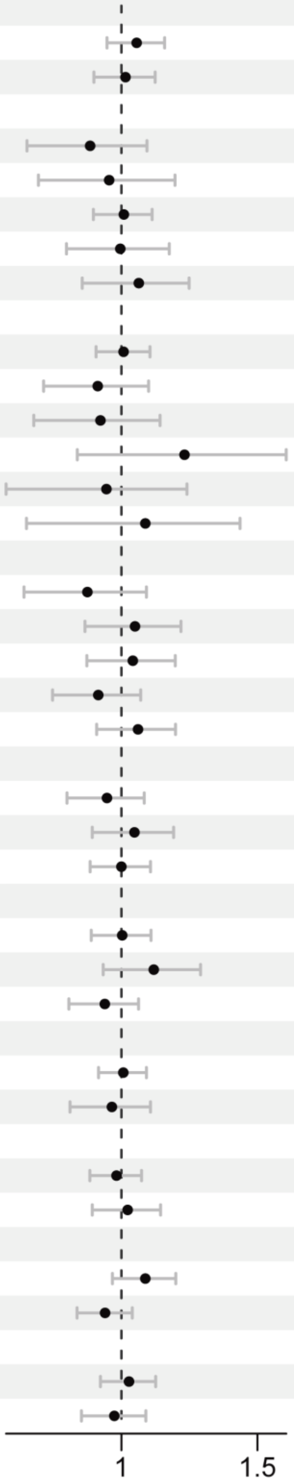

Supplementary Figure 8. Forest plot of stratified analysis of the association of blood cobalt ion concentrations on myocardial infarction.
